# Opportunities and Challenges in Gas Sensor Technologies for Accurate Detection of COVID-19

**DOI:** 10.3390/bios15120792

**Published:** 2025-12-02

**Authors:** Masoom Fatima, Munazza Fatima, Naseem Abbas, Pil-Gu Park

**Affiliations:** 1 Formerly at Department of Biology, Allama Iqbal Open University, Islamabad 44000, Pakistan; 2 Department of Microbiology, College of Medicine, Gachon University, Incheon 21936, Republic of Korea; munazzafatima@gachon.ac.kr; 3 Lee Gil Ya Cancer and Diabetes Institute, Gachon University, Incheon 21999, Republic of Korea; 4 Department of Mechanical Engineering, Sejong University, Seoul 05006, Republic of Korea; 5 Department of Life Science, Gachon University, Seongnam 13120, Republic of Korea

**Keywords:** gas sensor, VOC, exhaled breath analysis, COVID-19 detection

## Abstract

Gas sensors provide versatile opportunities for detecting volatile organic compounds (VOCs) such as acetone, methanol, ethanol, propanol, isoprene, and aldehydes in exhaled breath (EB) associated with COVID-19 respiratory infections. These VOCs provide valuable information about metabolic markers linked with COVID-19. They have opened opportunities to develop sensors for COVID-19 screening based on breath analysis. These sensors have the potential to provide the rapid detection of viruses in healthcare settings. RT-PCR, as a conventionally adopted diagnostic method, has a detection limit around 10–100 RNA copies/mL, with an accuracy of around 95%. Gas sensors have demonstrated VOC detection limits at the ppm level in COVID-19 EB and have displayed a sensitivity and specificity of 98.2% and 74.3%, respectively. Multiple gas sensors combined with machine learning algorithms have the potential to enhance the specificity of VOC detection. In addition to having an accuracy similar to that of the PCR method, the VOC-based diagnosis of COVID-19 offers unique advantages in terms of non-invasive and rapid detection. This review provides an overview of state-of-the-art gas sensors developed for COVID-19 detection. Despite there being significant developments in this field, there are certain challenges that still need to be addressed—these include the impact of environmental factors, the specificity of detection, the sensing range, and precision limitations, leading to accuracy issues. Despite these existing challenges, the integration of gas sensors with machine learning methods can enhance the accuracy of the detection of COVID-19. Future research directions are proposed to validate and standardize the application of gas sensors for COVID-19 in clinical settings.

## 1. Introduction

The SARS-CoV-2 coronavirus has affected millions of people across the globe since the beginning of the COVID-19 pandemic [1]. It poses a serious threat to elderly people and those with weak immunity due to diseases and health complications [2]. It has necessitated the development of advanced strategies for the early-stage detection of COVID-19 to improve global health [3]. Various diagnostic tools are applied in the detection of COVID-19—these include the reverse transcription polymerase chain reaction (RT-PCR), immunoassay-based antibody detection, imaging analysis, and exhaled breath analysis based on gas sensors. A brief description of these methodologies, with their pros and cons, is provided below. RT-PCR is the most well-known and widely accepted method for the reliable detection of COVID-19 infections [4]. It can detect single RNA molecules with higher accuracy [5]. While it has advantages relating to higher accuracy and reliable results, this approach has some drawbacks, including the requirement of high-purity samples and the use of expensive equipment. This can pose a particular challenge for resource-limited settings. Moreover, it also requires well-trained personnel [6], and it cannot be used to identify infections in their early stages. Immense effort has been devoted to developing diagnostic tools for the detection of COVID-19 as an alternative to PCR [7].

Particular attention has been paid to developing non-invasive and portable devices for the rapid detection of COVID-19 [8,9]. However, there are various challenges associated with achieving the detection of COVID-19 at a comparable accuracy to qPCR tests [10,11]. The RT-PCR method has been adopted as a standardized approach for COVID-19 diagnosis, having an LOD of about 10–100 RNA copies/mL while maintaining detection accuracies around 95% [12,13,14]. Gas sensors applied for EB of COVID-19 infections have shown detection limits of VOCs in the range of the ppm level while maintaining sensitivity and specificity of 98.2% and 74.3%, respectively [15]. Portable VOC detection approaches have offered alternative methods for exhaled breath analysis, resulting in satisfactory performance in clinical trials. These systems have integrated multiple gas sensors with machine learning algorithms, resulting in enhanced sensitivity and specificity of VOC detection. Devices such as GeNose C19 demonstrate consistent detection, with a sensitivity of 86–94% and a specificity of 88–95%. The accuracy of COVID-19 detection is gradually approaching that of RT-PCR [16,17,18,19]. However, there are major differences between the two approaches in terms of affordability and complexity of diagnosis. VOC-based detections, being a non-invasive approach and portable in nature without involving costly equipment, have emerged as an attractive strategy for rapid and affordable screening for COVID-19 infections.

In parallel, immunoassay-based techniques such as Lateral Flow Assays (LFA) and Enzyme-Linked Immunosorbent Assays (ELISA) have also been employed for COVID-19 detection by targeting specific antibodies or antigens. These offer the advantages of being affordable in cost and having a quick response. However, their sensitivity is less than that of PCR analysis. Furthermore, this approach may not be appropriate for early-stage detection of COVID-19. Moreover, changing viral loads during infection may affect the accuracy of the result [20]. Chest X-ray and computed tomography (CT) scan imaging analysis is applied for the diagnosis of COVID-19 infection. X-ray imaging is more cost-effective and easily approachable in limited-resource settings, whereas CT scans are comparatively more expensive and less readily available for mass diagnosis. Therefore, CT scans cannot be applied for large-scale diagnosis tests. X-ray analysis may not provide accurate detection at early stages of infection. Although CT Scan imaging analysis is an expensive approach, it offers the advantage of higher sensitivity [21,22].

Gas sensors for the detection of COVID-19 offer the advantages of low-cost approaches without requiring the involvement of costly equipment. This aspect is particularly useful for mass diagnosis, especially in resource-limited settings. Furthermore, their portable nature facilitates diagnosis with more ease. They provide rapid analysis, along with early-stage detection of COVID-19, which is not the case for the majority of existing methods conventionally adopted for the detection of infections [23,24]. A brief comparison of various diagnostic methods used for COVID-19 infections is summarized in Table 1. These parameters include limit of detection (LOD), sensitivity, selectivity, response time, and clinical studies to compare their capabilities for practical applications. Although traditional assays such as RT-PCR and immunoassays remain gold standards because of their high sensitivity and specificity, they have limitations of turnaround time and cost. In contrast, emerging biosensor and gas sensor platforms have shown a rapid response, portability, and suitability for applications in point-of-care or remote settings. Innovative technologies, including CRISPR-based assays and field-effect transistor (FET) biosensors, have ultra-sensitive detection capabilities with a high potential of clinical translation. All of these diagnostic methods collectively reflect the ongoing shift from laboratory testing toward accessible, real-time, and decentralized diagnostics.

**Figure 1:**
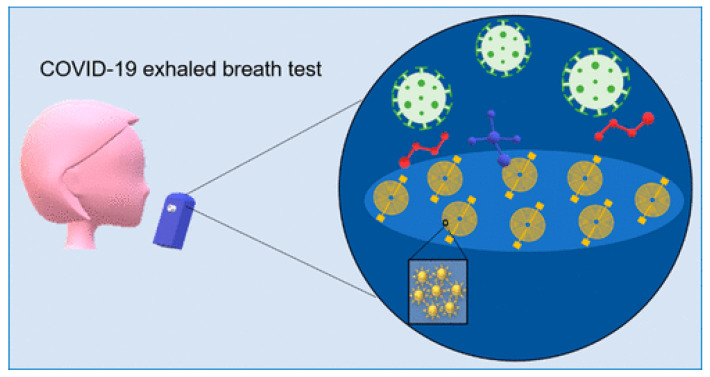
Schematic diagram of breath analysis for COVID-19 detection, adopted from reference [87].

Advancements in sensor technology are particularly useful for detecting COVID-19 infection [67,68]. Exhaled breath (EB), acting as a biomarker, provides useful information to diagnose COVID-19 [15,69,70] since long EB analysis is used for the diagnosis of various respiratory diseases [71]. Extending the concept of EB analysis to the detection of COVID-19 is relatively new, as it has emerged very recently [23,72]. VOC and exhaled gases existing in the breath can be detected by various methods, including FTIR [73], mass spectroscopy [74], electrochemical sensors [75], as well as gas chromatography [76]. Gas sensors for analyzing VOC in EB provide a simple and affordable solution for the detection of COVID-19 [77]. Semiconductor metal oxide, due to its gas detection ability, has shown promise for such applications [78,79,80]. Several gas sensors have been examined for their ability to precisely detect COVID-19 [81]. Schematics of EB-based detection of COVID-19 are shown in Figure 1. Since the breath composition of patients infected with COVID-19 can vary widely [82], achieving higher accuracy by avoiding false-positive and false-negative results remains challenging [83]. Integration of a machine learning algorithm with gas sensor technology has enhanced the accurate detection of COVID-19 [84]. Machine learning (ML) has recently emerged as an important tool to comprehensively analyze EB of patients infected with COVID-19. Commonly adopted sensors for various gases may lack higher specificity and sensitivity for EB analysis, particularly in the context of COVID-19 infections, considering the complexity and interferences. In this aspect, advancements in ML could be very useful to interpret and precisely differentiate various components of exhaled breath while maintaining higher detection accuracy [24]. Zou et al. have demonstrated the potential of AI-assisted breath analysis to detect VOC in COVID-19-infected patients with better sensing accuracy [18]. Similar findings have reported Random Forest (RF) and Support Vector Machine (SVM) algorithm-based VOC detections in COVID-19 detection using an electron nose configuration [17,43]. Such findings strongly reflect the significance of integrating ML as a potential strategy for reliable detection of VOCs in the exhaled breath of COVID-19 infected patients. The ML models, including conventional neural networks and gradient boosted trees have been integrated with spectroscopic methods for breath analysis to diagnose COVID-19 infections [85,86]. Such advancements in ML-based breath analysis could be a game changer in rapid, reliable interpretation of exhaled breath components, enabling a facile methodology in diagnostics of COVID-19 [42].

This review focuses on gas sensors limited to physiochemical detection of VOCs specific to COVID-19 infections only and does not cover biosensing platforms such as enzymes or antibodies. In this review, we have summarized the latest developments made so far to advance the gas sensor technologies for VOC detection related to COVID-19 infections. Furthermore, we have also summarized the existing challenges and proposed future research directions to make significant advancements in gas-sensor-based rapid and low-cost COVID-19 detection.

## 2. Biomarkers in EB for COVID-19 Detection

Biomarkers existing in EB provide valuable information about disease identification [88]. It provides a useful basis for non-invasive sensor-based detection of diseases through a facile diagnosis approach as compared to traditional methods. Breath analyzers have the advantages of a low cost, portable nature, and rapid detection. Human EB may contain about 3500 chemicals, including VOC and gases such as N_2_, H_2_, H_2_S, NH_3,_ and noble gases [89]. Among these EB, ammonia (NH_3_) is very important for the detection of kidney disease [90], renal failure [91], liver [92], and COVID-19 infection [93]. Recently EB analysis for detection of COVID-19 has gained significant attention [94]. The mechanism of NH_3_ formation in the body is as follows: when the kidney is not functioning properly, it may hinder the removal of urea. Urea accumulates in the blood, which is decomposed to NH_3_ and is responsible for raising its contents in the blood, skin, and EB [95,96]. Accurate detection of NH_3_ concentration in EB provides valuable information for kidney disease detection, especially chronic disease [97]. COVID-19 infection in certain patients could negatively affect multiple organs. Analyzing EB provides valuable information to track their health conditions. For instance, previous findings have reported that COVID-19 patients with kidney diseases have shown elevated levels of blood urea nitrogen (BUN) contents [98]. Narasimhan et al. have demonstrated a correlation between BUN and NH_3_ in EB among dialysis patients. They demonstrated that NH_3_ in EB decreased significantly after dialysis, establishing a strong connection between BUN and NH_3_ [99]. This shows that measuring NH_3_ in EB using gas sensors could be very supportive in diagnostics. However, there could be certain challenges associated with such detection, including interference of other substances and challenges to detect lower concentrations of NH_3_ < 50 ppb [100].

**Figure 2:**
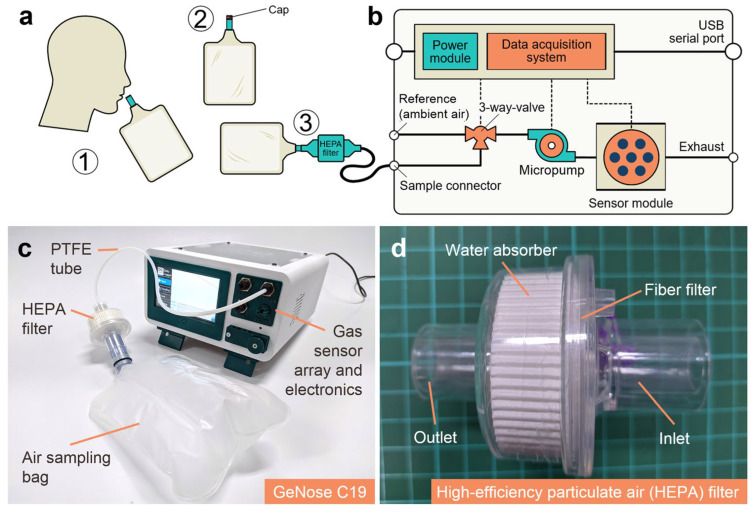
Scheme of GeNose C19, (**a**) breath sampling pipeline: (1) air inhaled through the nose, (2) sealing of sampling bag, (3) plugging of the sampling bag into the electronic nose inlet; (**b**) diagram and (**c**) photograph of GeNose C19 and (**d**) HEPA filter for filtering out the particulate matters and trapping the SARS-CoV-2 available from the exhaled breath adopted from reference [17].

The working principle of gas sensors for COVID-19 detection is to analyze various components of EB, which enables the detection of COVID-19 infection. Various types of gas sensors can facilitate identification of VOC patterns in EB. In this non-invasive analysis, as the gaseous components of EB interact with the surface of gas sensors, it leads to changes in the electrical resistance. This is associated with an increase or decrease in output voltage. Alteration in electrical resistance is the most significant characteristic of sensors, which is helpful in distinguishing healthy persons from COVID-19-infected patients. In contrast, healthy individuals do not show considerable changes in electrical resistance during interaction with the sensing platform based on analysis of EB. The change in resistance is translated into electrical signals. An integrated setup for gas sensing integrated with a data acquisition setup is shown in Figure 2.

**Figure 3:**
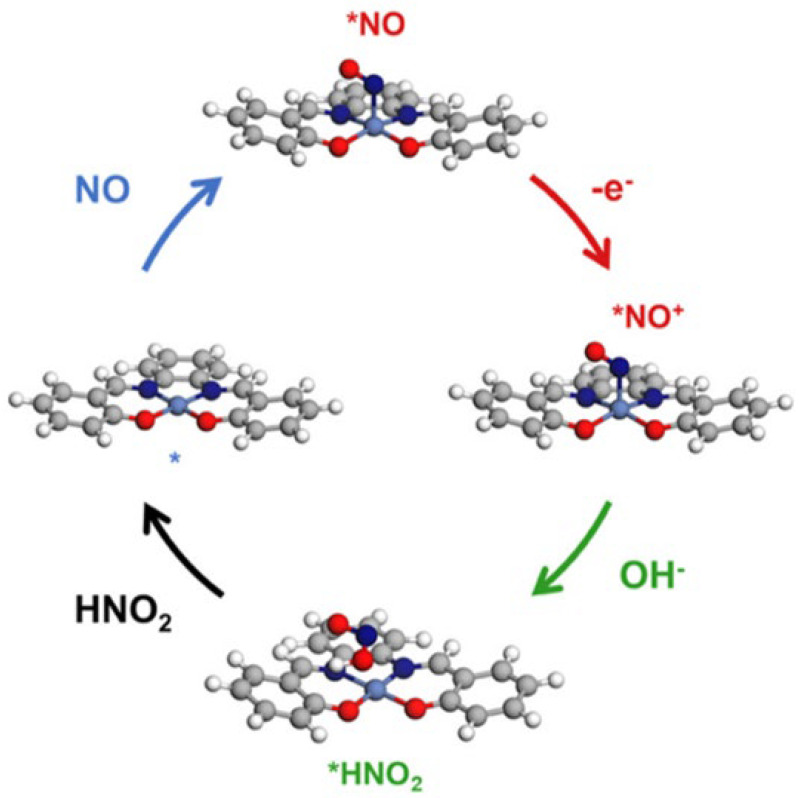
The mechanism for one e^–^ NO electrocatalytic oxidation for the sensing of NO gas adopted from reference [101].

NO gas is an important biomarker in EB of COVID-19-infected patients. The underlying mechanism involving NO sensing is related to the oxidation of NO gas. The pathway involved in the oxidation of NO is highlighted by various findings [101,102,103]. First, NO is adsorbed at the active site of the sensor (*NO), followed by one electron transfer from *NO to the sensor and the production of *NO^+^. This intermediate is readily oxidized in the presence of OH^-^ to produce HNO_2_ [101]. The change in NO concentration at the surface of the sensor leads to a change in resistance of the sensor, leading to its detection. Various stages involved in the oxidation of NO are highlighted in Figure 3.

## 3. Advancements in Gas Sensors for COVID-19 Detection

**Figure 4:**
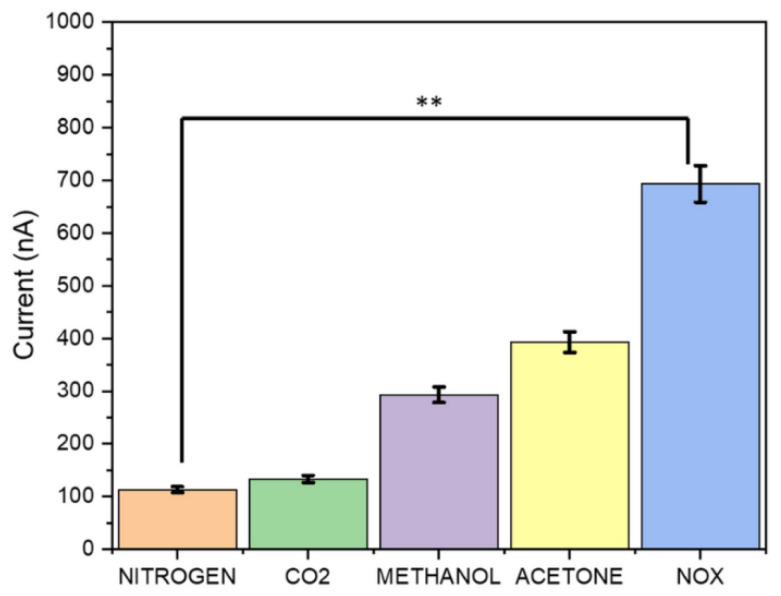
Sensing ability of a fabricated sensor to detect various gases in EB, adapted from reference [104].

Extending the gas sensing technology to the detection of COVID-19 has emerged recently. The latest findings in this field are summarized as follows. Zhou et al. have designed a single-nickel-atom-based sensor for screening for COVID-19. This sensor can detect fractional exhaled nitric oxide (FeNO). It has shown highly sensitive detection of NO within the range of 0.3–180 ppb. Their finding has shown a strong correlation between FeNO and COVID-19 infection. Integrating FeNO with a machine learning model has facilitated differentiating healthy persons from COVID-19 patients. This work demonstrates a highly sensitive exhaled NO sensor for the detection of COVID-19 in a simplified and affordable approach [101]. Banga et al. have demonstrated a breath-analyzer-based sensor for the detection of NO gas in the range of 50 to 250 ppb. They have exploited the use of room temperature ionic liquid (RTIL) for accelerating the diffusion of NO gas. 1-Ethyl-3-methylimidazolium Tetrafuoroborate ([EMIM]BF_4_) has been used as an IL. The amount of electrical current generated in the sensor was used to distinguish between negative and positive COVID-19. This work demonstrates the significance of ionic liquids to improve the NO sensing ability of the sensor, which has enabled the rapid detection of COVID-19 in a portable prototype design [104]. The sensitivity of the sensor against various gases shows that the highest performance was observed for NO detection (Figure 4).

**Figure 5:**
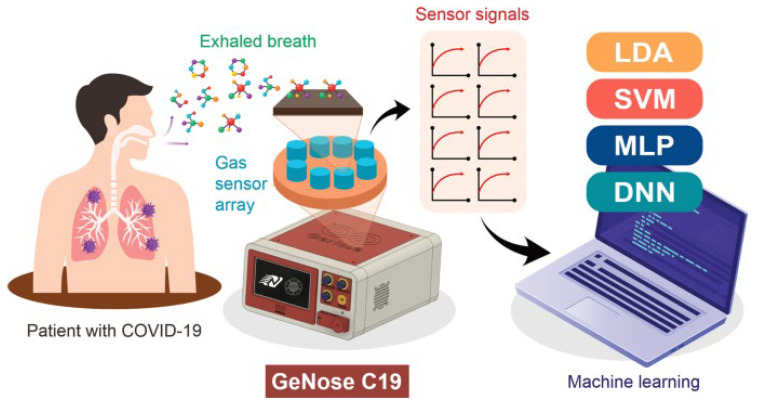
Schematics for integration of a portable electronic nose (GeNose C19) with AI, adapted from reference [17].

Advancements in AI and machine learning have been applied recently for the analysis of EB. The combination of gas sensors with a machine learning model is presented in Figure 5.

**Figure 6:**
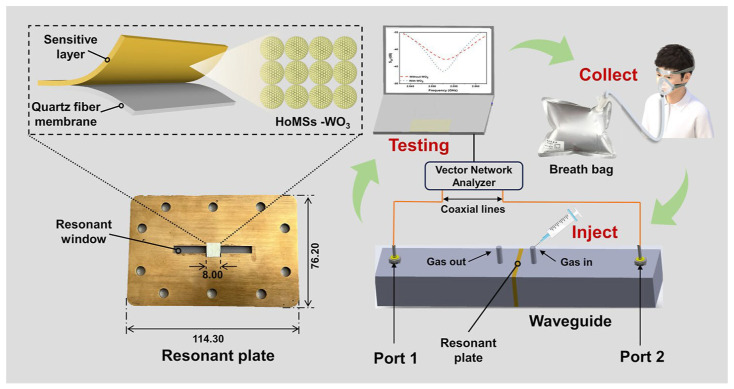
Schematic diagram of WO_3_-based gas sensor, adapted from reference [106].

WO_3_ has demonstrated the capability to detect NO and NH_3_ gases [105]. Wang et al. have designed a WO3-based microwave gas sensor (MGS) to detect NO in EB for the diagnosis of COVID-19. WO_3_ nanostructures were composed of multi-shelled hollow configurations, which were prepared by adopting template-assisted synthesis. The fabrication of the WO3-based waveguided sensor for COVID-19 detection, and its working principle is shown in Figure 6. These WO_3_ nanostructures were loaded on a quartz fiber membrane to make a sensor. A higher selectivity was observed for the detection of NO in the range of 10–100 ppb. A strong correlation was observed between NO concentration and COVID-19 detection [106]. It has been challenging to detect COVID-19 in critically ill patients. Exline et al. have demonstrated a breadth analysis of such patients from the intensive care unit (ICU) for disease detection. Catalyst-based sensors were applied to detect NO and NH_3_ gases from breath. EB was rapidly analyzed in a short duration of 15 s. The sensor adopted for breath analysis was made from WO_3_ nanocrystals prepared by the sol–gel method. A thin film of catalyst was fabricated and integrated with a breath analyzer to detect the NO biomarker in EB. The sensor developed in this work has shown interesting findings with 88% accuracy. However, more improvement in accuracy would be required to achieve an accuracy level closer to the qPCR test [107]. WO_3_ sensors have demonstrated extended stability for the detection of NO_2_ gas due to its stable structure. This reflects that use of metal oxides could be applied as a stable sensing material for detection of COVID-19 [108].

**Figure 7:**
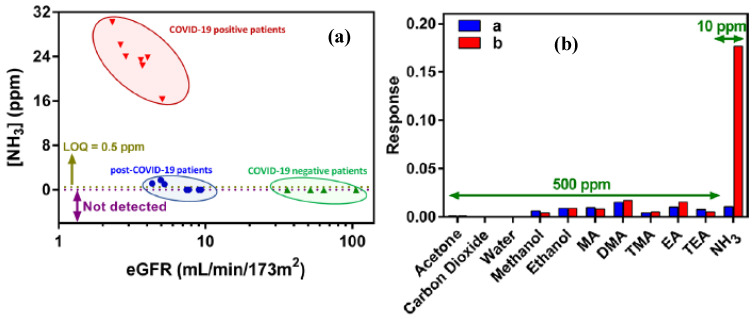
Responses of the (**a**) Au-IDEs/S-PPyMPs and (**b**) Au-IDEs/S-PPyMPs/AgNPs sensors toward 500 ppm of different interfering compounds and 10 ppm of NH_3_; (**b**) adopted from reference [93].

Kamalabadi et al. have focused on designing a novel chemiresistor gas sensor for detection of NH_3_ in EB of COVID-19 patients suffering from acute kidney injury (AKI). The sensor was fabricated as a thin film of polypyrrole/silver nanoparticles integrated with gold interdigital electrodes (Au-IDEs/S-PPyMPs/AgNPs). It exhibited NH_3_ detection in the range of 1.00−19.23 ppm. Applying this sensor for COVID-19 patients has demonstrated encouraging accuracy for NH_3_ detection. Figure 7a shows that NH_3_ content in COVID-19 was significantly higher as compared to healthy individuals. The designed sensor has shown high sensitivity for NH_3_ as compared to other compounds such as CO_2_, acetone, ethanol, and methanol. A highly intense response was noticed even with a lower concentration of NH_3_ (10 ppm), but other compounds with a much higher concentration (500 ppm) have shown negligible signal intensity (Figure 7b). This reflects a higher selectivity of the sensor for NH_3_ detection and minimal interference from other compounds. The incorporation of Ag NP in Au-IDEs/S-PPyMPs has particularly enhanced the sensitivity. It has resulted in 13-fold improvement in the signal as compared to pristine Au-IDEs/S-PPyMPs. This study demonstrates a simplified non-invasive approach based on NH_3_ detection for COVID-19 patients suffering from kidney disease [93]. Shan et al. have fabricated a multiplexed nanomaterial-based sensor for the detection of COVID-19 through EB analysis. The electrodes were prepared by photolithography on a silicon wafer. Gold nanoparticles (GNP) coated with organic ligands were printed on the electrodes. The designed sensor was applied to monitor VOC biomarkers in EB of COVID-19 infection. Sensor operation was based on detecting the change in its electrical resistance on exposure to VOCs in EB. Clinical investigations were carried out by comparing the sensor response for COVID-19-infected patients, as well as control samples of non-infected individuals. It has demonstrated accurate analysis to distinguish between infected and non-infected individuals. Results obtained by using this approach were comparable to the conventionally adopted RT-PCR method.

This finding highlights the implication of nanoparticle-based systems to detect COVID-19 with higher sensitivity [87]. Nurputra et al. have developed an electronic-nose-based sensor (GeNose C19) for VOC analysis in EB to detect COVID-19 as an alternate to RT-qPCR. They have integrated a machine learning algorithm with gas sensors to accurately analyze EB. The use of metal oxides as a sensing material provides the benefit of a low cost due to their natural abundance. The sensing unit works on the principle of chemoresistivity analysis. The breath sampling unit has been designed carefully to avoid interference and leakage to the surrounding environment. During the test, when VOC interacts with the sensor, it reduces the electrical resistance in a short time (40 s). It enables the operation of GeNose C19 in a shorter sensing duration. The diagnostic tool developed in this work, however, may require preheating to stabilize the signal. Although EB composition may vary from patient to patient, GeNose C19 has demonstrated precise detection of negative and positive COVID-19 patients; results were quite similar when cross-checked with RT-qPCR. This finding highlights the significance of exploiting metal oxide as a low-cost material for EB analysis and demonstrates its integration with machine learning models to accurately diagnose COVID-19 [17]. In another finding, Bhaskar et al. have integrated machine learning models for the detection of COVID-19 through analyzing VOCs such as acetone, methanol, isopropanol, and ethanol in EB. The sensing chamber has four sensors to detect these biomarkers. Gas sensors have been integrated with neural-network-categorical boosting (CNN-CatBoost) for the detection process. It has demonstrated a satisfactory accuracy of 96.15% to detect COVID-19. They have explored the metal-oxide-based sensors TGS1820, Alc/C100, MQ3 to detect acetone, methanol, and ethanol, while a commercial detector by ALVI Automation Pvt. Ltd. was adopted to detect isopropanol. Variation in gas concentration was linked to changes in electrical conductivity, which provided the basis of biomarker detection. Clinical investigations have confirmed a high accuracy of the designed setup for the detection of COVID-19 [109]. Kwiatkowski et al. have performed clinical trials for EB analysis to detect COVID-19 by applying an electronic nose. In this finding, COVID-19 patients, healthy individuals without infection, and air samples were analyzed for comparison to interpret the accuracy of the analysis. A Teflon-made breath sampler was employed to avoid contamination. The electronic nose was composed of commercially available gas sensors. The change in DC resistance on exposure to VOC gases was translated for disease detection. The neural network and Random Forest algorithms reflected an 84% accurate analysis in differentiating healthy and infected individuals [40]. Bellarmino et al., in another finding, briefly analyzed VOCs in EB and data analysis, which was assisted by implementing machine learning algorithms. They have performed clinical trials to investigate EB analysis assisted by a mass spectrometer (MS) and observed accuracy in detection. Breath samples were collected with the help of a straw attached to the collection bag, which was further transferred to MS to analyze the m/z of EB components. Although the approach adopted in this study is not in real time, it provides valuable insight about fingerprinting of VOC in EB and COVID-19 detection through an integrated approach of exploiting machine learning and mass spectroscopy [24]. Aguilera et al. in their recent work highlighted the application of magnetic (Fe_3_O_4_) nanoparticles for the real-time detection of breath biomarkers using a wireless sensing platform. The sensor operates on the principle of magnetoelastic resonance shifts, where interactions between VOCs in EB and the magnetic nanoparticles alter the sensor’s magnetic permeability and resonance frequency. These variations are wirelessly monitored to provide real-time, non-invasive analysis of exhaled biomarkers. The study demonstrates that changes in the magnetization of nanoparticles can be directly correlated with VOC concentrations, offering valuable insight for disease detection. The current findings highlight the strong potential of magnetic nanoparticle-based sensors to extend their application toward VOC biomarker analysis for COVID-19 detection and other respiratory diagnostics [110]. Sharma et al. have developed a portable breath-based VOC monitoring system integrated with a sensor array for the rapid detection of COVID-19 infection. Their study employed portable gas chromatography combined with machine learning algorithms to analyze VOC profiles in EB and identify COVID-19-specific signatures. They further evaluated the system’s diagnostic accuracy across emerging SARS-CoV-2 variants, including Delta and Omicron, emphasizing the need for continuous model retraining and calibration as viral mutations alter VOC compositions. This finding highlights that diagnostic tools must not only be capable of distinguishing between healthy and infected individuals but should also be able to detect metabolic variations introduced by different viral strains. Ensuring the precise detection of variant-specific VOC biomarkers is therefore essential for maintaining diagnostic reliability. Moreover, this approach provides a foundation for the future design of portable, adaptive gas sensors capable of detecting multiple COVID-19 variants and other respiratory infections [111]. Zou et al. have employed a machine-learning-assisted gas sensor array to detect COVID-19 by analyzing VOCs present in EB. Their study integrated multiple gas sensors with artificial neural networks (ANNs) and other machine learning models, such as Support Vector Machines (SVMs) and Principal Component Analysis (PCA), to achieve precise pattern recognition and classification of sensor responses. The researchers compared the performance of lab-fabricated sensors with commercial sensor arrays for the detection of nine representative VOCs associated with COVID-19. The AIST-developed sensors exhibited superior sensitivity and selectivity compared to commercial counterparts, demonstrating the potential of optimized sensor–ML integration for accurate, rapid, and non-invasive COVID-19 detection. These findings underscore the effectiveness of data-driven sensor optimization in enhancing the diagnostic accuracy of breath-based biosensing systems [18]. A summary of biosensors adopted for EB analysis is presented in Table 2.

## 4. Summary and Future Perspectives

Recently, substantial developments have been made in fabricating gas sensors for the detection of COVID-19. Innovations in materials and machine learning have encouraged the design of advanced sensor technology. It has facilitated the improved analysis of various biomarkers, including NO, NH_3_, and VOCs, in EB to determine COVID-19 infection. Gas sensors, being non-invasive and advanced diagnostic tools, have provided encouraging outcomes. Real-time application of sensor technology for COVID-19 detection can emerge as an affordable and portable diagnostic tool. This technology has the potential to replace the existing time-consuming and expensive qPCR analysis techniques. However, for practical applications of gas sensors for COVID-19 detection, there are still certain challenges and limitations. These include alternation in breath profiles based on viral mutations and the emergence of new strains. This could potentially cause interference in the analysis. In order to avoid such interferences, it is important to carefully monitor the strain-specific response of gas sensors [111]. Moreover, gas sensors are lacking validation and standardization protocols for indoor environments. This raises concerns regarding the establishment of reliable diagnostics for the precise detection of gases in breath analysis. Sensors opted for VOC analysis associated with COVID-19 could be influenced by environmental factors such as temperature. It is important that sensors maintain a reliable response under different circumstances [112]. Furthermore, the selectivity of the sensor should not be affected by the complex composition of EB. New developments in gas sensors for COVID-19 detection are showing promising directions for further research. Integrating gas sensors with machine learning could pave the way for enhancing accuracy for the detection of COVID-19 through breath analysis [113]. Innovation in nanomaterials with ultra-high sensitivity could facilitate the fabrication of gas sensors with the enhanced detection of trace biomarkers in EB. Furthermore, in the future, efficient biosensors should be designed that can detect multiple biomarkers without compromising the accuracy of detection. This rapidly evolving technology could be extended for the detection of other infectious diseases to improve public health using low-cost and affordable diagnostics.

The advent of gas sensors for exhaled breath analysis has opened new opportunities for the diagnosis of COVID-19. Compared to other diagnostic tools, the following are the pros and cons of gas sensors. Gas sensors are a non-invasive approach for sampling. Sampling required for the diagnosis of COVID-19 through PCR and immunoassay analysis could be inconvenient for infected individuals. Similarly, CT scans and X-ray imaging may require visiting specialized testing facilities for diagnosis. However, simplified exhaled breath analysis based on gas sensors presents a simplified strategy for diagnosis. Portable nature highlights the potential of gas sensing technology to extend mass diagnosis, especially in rural areas and resource-limited settings. Moreover, unlike PCR- and ELISA-based methods, the gas sensors provide rapid detection, delivering results in a short time. Gas sensors for COVID-19 do not involve the use of expensive equipment, enabling them to be a low-cost methodology that is affordable and readily available to a broader population, especially in low-income settings. Such features make gas sensors an attractive alternative for COVID-19 diagnosis. The accuracy of gas sensors is lower compared to PCR and immune assay methods. However, these methods require sufficient viral load for the diagnosis of COVID-19. This is not the case for gas sensors; they can be applied for early-stage detection of COVID-19. Such advantages of gas sensors are very important for preventing the spread of infection to healthy individuals by taking timely measures, such as isolation and the adoption of treatment measures. Despite their great potential discussed above, several challenging directions remain that could hinder the broader application of gas sensors for VOC detection of COVID-19. Since the quality of EB could greatly vary from person to person, certain parameters such as lifestyle, age, viral load, and the co-existence of other infections may have a significant impact on VOC composition in EB analysis. This can cause possible interference or false results. Furthermore, since this research direction is still at an initial stage, it lacks a standard for the authentication of results. In certain cases, the accuracy of these detections is somewhat lower than that of widely practiced PCR diagnosis. There are also specificity challenges; gas sensors may lead to an inaccurate detection when exposed to breath analysis of individuals infected with other respiratory infections. Addressing the above challenges may have a significant impact on improving the performance and accuracy of gas sensors for the diagnosis of COVID-19. It will extend their applications at a wider scale for precise diagnosis. Finding a solution to these challenges can also be useful for extending their application to the diagnosis of respiratory infections beyond COVID-19.

## 5. Conclusions

Analyzing volatile organic compounds in exhaled breath analysis through gas sensors is emerging as a versatile and reliable diagnostic tool for the detection of COVID-19. As a non-invasive and simple approach, it offers competitive advantages as compared to the conventionally adopted PCR method for the detection of COVID-19. It does not require the use of complicated equipment and provides rapid detection, which is crucial in the rapid screening of infections. Metal oxides in this regard have been offered as an attractive platform, ensuring the accuracy of detection in the range of 85–95%, being quite comparable to PCR-based detection. The integration of machine learning algorithms with gas sensing has shown potential for reliable detection of VOCs in the EB of COVID-19 infections. Besides impressive developments made so far in this field, there are existing challenges that restrict the widespread use of gas sensor technology for the diagnosis of COVID-19. Owing to such limitations, gas sensors are facing barriers in the transition from lab-scale prototype to approved diagnosis. Existing challenges include a lack of fingerprinting in the identification of VOC patterns specific to COVID-19. There is a probability of interfering VOC substances in EB originating from diseases other than COVID-19. It is needed to design gas sensors for VOCs analysis of COVID-19 detection, having high specificity and minimal interference from other substances. Ongoing investigations made so far are in their infancy and mainly limited to the academic scale. Rigorous efforts are needed to approach the clinical trials of these sensors, expanding them to large populations to map their effectiveness for real-time point-of-care COVID-19 diagnostics. Regulatory approvals and standardization of gas sensors for VOC detection of COVID-19 infections are still lacking. Furthermore, low-cost sensors with reproducible detection capabilities should be developed, considering the practical applications. Therefore, it is highly desired to address the above-mentioned challenges to establish the availability of this platform for real-time detection of VOCs in EB of COVID-19 infections.

## Figures and Tables

**Table 1:** Summary of diagnostic methods for the detection of COVID-19.

Method	Target Analyte	Detection Limit	Response Time	Sensitivity	Specificity	Cost	Settings	Clinical Validation Status	Reference
RT-PCR	RNA	~10–100 copies/reaction	≈1 h 30 min	98.1–100%	95.7–100%	expensive	Labs	extensively validated, globally approved	[4,12,25,26,27,28]
Immunoassay/(Antigen/Antibody)	Viral antigen/host antibodies	~10–100 ng/mL	30 min	56.2%	99.5%	Low cost	Point of care	Clinically validated, approved	[29,30,31,32,33,34]
Chest X-ray /CT Imaging	Lung abnormalities	N/A	Rapid	44–98%	25–96%	Moderate	Hospitals & Labs	Clinically validated for monitoring	[35,36,37,38]
Gas sensors for exhaled breath	VOCs and gases	ppm–ppb range	Rapid	98.2%	74.3%	moderate	portable	Limited pilot-level clinical validation	[15,17,24,39,40,41,42,43]
QCM biosensor	Viral particles	40–210 pfu/mL	≈5 min	98.15%	96.87%	Moderate	Research labs/pilot clinical	Early clinical validation, under evaluation	[44,45,46]
SERS biosensor	Spike protein/viral components	≈300 nM	5 min	95%	95%	Moderate–High	Lab validation	Research-stage validation, not yet approved	[47,48,49,50,51,52]
FET/BioFET	Viral proteins/RNA	pM–fM	>1 min	Ultra-sensitive	Ultra specific	Moderate–High	Lab/small-scale clinical	Limited clinical validation, development ongoing	[53,54,55]
CRISPR-Cas Diagnostics	Viral RNA	~10 copies/µL	~15–60 min	94%	98%	Moderate	Point-of-care/low resource	Clinically validated, several approved assays	[18,56,57,58,59,60,61]
CRISPR-MCDA one-pot	Viral RNA	~10 copies/µL	30–45 min	96–98%	100%	Moderate	Lab/near POC	Pilot-level clinical validation, limited regulatory approval	[61,62]
Wearable Sensor Systems	Physiological biomarkers	N/A	Continuous	36.5–100%	73–95.3%	Low-Moderate	Remote/consumer	Clinically studied, limited diagnostic approval	[63,64,65,66]

**Table 2:** Summary of gas sensors for EB analysis to detect COVID-19.

Sensor	Gas/VOC	Detection Limit	Accuracy	Reference
Nickel based SAC	FeNO	1.8 nM	-	[101]
[EMIM]BF_4_	NO	50 ppb	91.36%	[104]
HoMSs-WO_3_	NO	2.52 ppb	-	[106]
WO_3_	NO	-	85%	[107]
Au-IDEs/S-PPys/AgNP	NH_3_	0.12 ppm	-	[93]
CNN-CatBoost model	Acetone Ethanol Methanol Isoproponol	-	98.97–99.04%	[109]
Fe_3_O_4_	Acetone	0.75 ppm	-	[110]
Oxide semiconductor	VOCs	-	90–95%	[87]

## Abbreviations

The following abbreviations are used in this manuscript:

VOC	volatile organic compounds
qPCR	quantitative polymerase chain reaction
NH_3_	ammonia
BUN	blood urea nitrogen
FeNO	fractional exhaled nitric oxide
MOS E-nose	metal oxide semiconductor electronic nose
QCM biosensor	quartz crystal microbalance biosensor
SERS biosensor	surface-enhanced raman scattering biosensor
FET/BioFET	field-effect transistor/biological field-effect transistor
AUC	area under the curve.
pM–fM	picomolar to femtomolar
CNN	convolutional neural network
SVMs	support vector machines
COVID-19	coronavirus disease 2019
RT-qPCR	reverse transcription quantitative polymerase chain reaction.
